# Long-Term Prescription of α-Blockers Decrease the Risk of Recurrent Urolithiasis Needed for Surgical Intervention-A Nationwide Population-Based Study

**DOI:** 10.1371/journal.pone.0122494

**Published:** 2015-04-13

**Authors:** Chia-Chu Liu, Hui-Min Hsieh, Chia-Fang Wu, Tusty-Jiuan Hsieh, Shu-Pin Huang, Yii-Her Chou, Chun-Nung Huang, Wen-Jeng Wu, Ming-Tsang Wu

**Affiliations:** 1 Department of Urology, College of Medicine, Kaohsiung Medical University, Kaohsiung City, Taiwan; 2 Department of Urology, Kaohsiung Medical University Hospital, Kaohsiung Medical University, Kaohsiung City, Taiwan; 3 Department of Urology, Pingtung Hospital, Ministry of Health and Welfare, Executive Yuan, Pingtung, Taiwan; 4 Department of Public Health, Kaohsiung Medical University, Kaohsiung City, Taiwan; 5 Department of Medical Genetics, College of Medicine, Kaohsiung Medical University, Kaohsiung City, Taiwan; 6 Department of Family Medicine, Kaohsiung Medical University Hospital, Kaohsiung Medical University, Kaohsiung City, Taiwan; 7 Center of Environmental and Occupational Medicine, Kaohsiung Municipal Hsiao-Kang Hospital, Kaohsiung Medical University, Kaohsiung City, Taiwan; Sun Yat-sen University, CHINA

## Abstract

**Purpose:**

α1 receptors and subtypes have been confirmed to distribute in human pelvis and calyces recently. As used in ureteral stones, α-blocker treatment may facilitate kidney stone passage and long-term prescription of α-blocker may decrease the risk of recurrent urolithiasis. The aim of this study is to determine if use of α-blockers 180 days or more can decrease the risk of recurrent urolithiasis needed for surgical intervention.

**Materials and Methods:**

A representative database of 1,000,000 patients from Taiwan’s National Health Insurance was analyzed. Eligible patients were those who had received the first-time procedure for upper urinary stone removal, including extracorporeal shock-wave lithotripsy, ureterorenoscopic lithotripsy, or both, between 2000 and 2010. After completing a 180-day treatment for first event, patients were prospectively followed-up until a second set of stone procedures was performed (proxy of stone recurrence), loss to follow-up, or end of study. The effect of percentage of total number of days of α-blocker use on need for second set of stone procedures within a post treatment 180-day follow-up period was analyzed by quartile. A nested case-control study was also performed.

**Results:**

1,259 patients were eligible for final analyses. During 3,980 person-years follow-up, 167 patients had recurrent urolithiasis needed for surgical intervention. From first to fourth quartile of drug exposure, recurrence rates were 45.64, 47.19, 43.11, and 18.52 per 1,000 person-years. The adjusted hazard ratio was 0.46 (95% CI = 0.24 to 0.89) for the fourth quartile (vs. quartile 1). In the nested case-control study, adjusted ORs was 0.23 (95% CI = 0.10 to 0.53) in the fourth quartile (vs. quartile 1). The results remained similar even in patients categorized by cumulative defined daily dose (cDDD) quartiles and average cDDD per day quartiles.

**Conclusion:**

Use of α-blockers for 180 days or more decrease the risk of recurrent urolithiasis needed for surgical intervention. In patients at higher risk of recurrent urolithiasis, long term prescription of α-blockers might help them prevent further surgical intervention.

## Introduction

Urolithiasis, urinary tract stones, affects almost all populations across different regions, cultures, and races [1–2]. It is associated with various comorbidities and increased risk of chronic kidney disease, metabolic bone disease and cardiovascular events [3]. The lifetime risk is 10–25% around the world [1,4]. After treatment, urolithiasis recurs within 5–10 years in ~50% of patients and in 75% within 20 years [1,5]. This disease has steadily increased in incidence and prevalence worldwide, prompting the need for primary and secondary prevention [2].

While many patients remain asymptomatic, others have pain, urinary tract obstruction, infection, or loss of renal function. Patients often need to visit emergency departments and phycisians’ clinics to receive surgical interventions. Around 1.0%-1.7% of all emergency department visits in the United States are for renal colic or urolithiasis [6]. The economic burden of urolithiasis is immense. According to data from the Urological Diseases in America Project, the direct and indirect cost of treating urolithiasis in the United States in the year 2000 was about $5.3 billion [7].

Although invasive surgical interventions have been minimized and replaced with extracorporeal shock-wave lithotripsy (ESWL) or ureterorenoscopic lithotripsy (URSL), complications and high costs of treatment remain [7]. Several medications have been found to possibly facilitate the passage of stones and reduce their recurrence [8]. One, α-adrenoreceptor antagonist (α-blocker), is reported to augment stone expulsion rates, reduce the time to expulsion, and lower analgesia requirements for ureteral stones with and without surgical intervention [9,10]. Recently, the presence and distribution of α1 receptors and subtypes in human pelvis and calyces has been confirmed by Karabacak et al [11]. Their findings imply that α-blocker treatment could facilitate kidney stone passage and help decrease pain, as used in ureteral stones [11]. In addition, long-term prescription of α-blockers may also decrease the risk of recurrent urolithiasis.

However, clinical trials have thus far only examined the effectiveness of α-blockers on stone clearance before or after such surgical interventions as ESWL over a short period of time (<90 days) [9,10]. Whether longer periods of treatment with α-blockers can prevent the recurrence of stones and decrease the necessity of further surgical intervention over longer periods is still not known. To find out, this study used a nationwide representative population-based dataset to study the effect of follow-up α-blocker treatment on stone recurrence needed for surgical intervention in patients who previously completed a full treatment course for urolithiasis. The percentage of number of dosage days (out of 180 days), cumulative defined daily dose (cDDD), and average cDDD per day were analyzed in a retrospective cohort study and nested case-control study to investigate the effect of dosage on recurrence.

## Materials and Methods

### Data Sources

Longitudinal sampling cohort data from the National Health Insurance Research Database (NHIRD) between January 01, 1999 and December 31, 2010 was used for analyses. For research purposes, Taiwan’s National Health Research Institutes (NHRI) created randomly sampled representative database of 1,000,000 patients from the year of 2000 registry of all NHI enrollees using a systematic sampling method. According to NHRI, there are no significant differences in age, sex, or health care costs between the sampled group and all enrollees [12].

### Characteristics of the NHIRD

This dataset contains comprehensive demographic characteristics such as gender, date of birth, and income levels, and health care data including dates of outpatient visits and inpatient admissions or discharges, clinical diagnoses (up to five coexisting diagnoses), medical procedures (up to five diagnostic or therapeutics procedures), NHIRD internal billing codes (S1 Table), and detailed drug prescription information (i.e., names of prescribed drugs, dosage, date of prescription). The codes of clinical diagnoses and procedures used in this database (S1 and S2 Tables) are the same as those used by the International Classification of Diseases, Ninth Revision, Clinical Modification (ICD-9-CM) [13]. This study was approved by the Institutional Review Board of Kaohsiung Medical University Hospital. Since the dataset contains aggregated secondary data and the patient identifiers are scrambled to the public for research purposes to protect confidentiality, the requirement for written or verbal consent from patients for data linkage study was waived. The protocol for this study conforms to the ethical standards set forth in the Declaration of Helsinki 1964.

### Study Cohort

**Fig 1:**
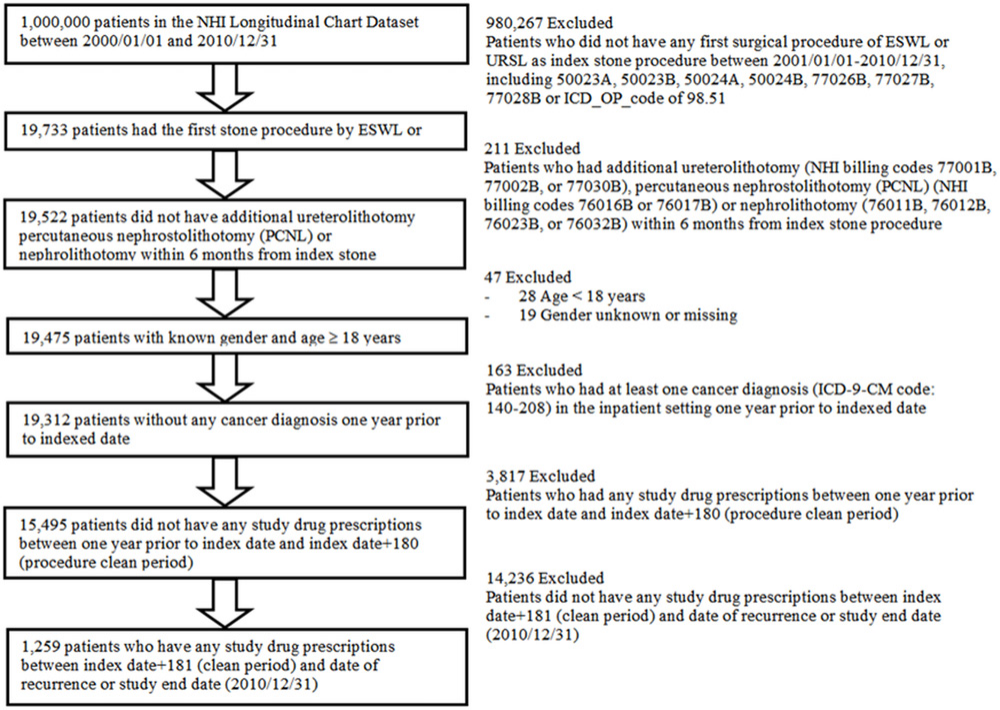
Study flowchart.

The potential subject for this study were patients with claims for first-time upper urinary tract stone removal procedures either by ESWL, URSL, or both in either outpatient or inpatient settings between January 01, 2000 and December 31, 2010 (Figs 1 and S1). This first surgical stone removal procedure was defined as index stone procedure. The 180-day period following each index procedure was consider part of the first treatment to be confirmed completely. This time period was chosen because the average time period within which upper urinary tract urolithiasis can be resolved by ESWL, URSL or both is reported to be less than 90 days in Taiwan [14,15].

Potential patients were excluded if they had an additional ureterolithotomy, nephrolithotomy or percutaneous nephrostolithotomy (PCNL) within 180 days of index stone procedure. Those patients may have complex urolithiasis with large stone burden or anatomic abnormality and their etiology and management may be different from those of most urolithiasis patients. Additional exclusion criteria included a patient being under 18 years old, not knowing the gender of the patient, having an index procedure on the same day as the end of study date, and a patient having a diagnosis of cancer (ICD-9-CM diagnosis codes between 140 and 208) in the inpatient setting one year prior to index date. We used the new user design to identify the exposed patient group who were not prescribed any α-blocker medication between one year prior to index date and the last date of the complete treatment period [16]. Finally, eligible patients were those who were prescribed any of the study drugs 180 days after the index date (Fig 1).

### α-blocker Medications

α-blocker medications, including tamsulosin, terazosin, doxazosin, and alfuzosin, were the major study drugs of interest [16–18]. We collected the dates of prescriptions, the daily dose, the number of days dispensed, and the number of pills per prescription. To minimize potential time-related bias, a 180-day drug exposure window was used to calculate percent of prescribed days within 180 days from the date of first pharmacy claim after the 180-day complete treatment period (S1 Fig) [17]. In order to evaluate the overall effects of study α-blocker medications, the defined daily dose (DDD) was used for each study α-blocker (S3 Table). The defined daily dose (DDD) is recommended by the World Health Organization (WHO) for a unit to measure a prescribed amount of drug. It is assumed the averaged maintenance dose per day of a drug consumed for its main indication in adults. Through standardizing daily doses, different drugs can be compared given same standard unit [19]. Cumulative defined daily dose (cDDD) and average cDDD per day in each group were calculated. cDDD was calculated as the sum of dispensed DDD within the drug exposure time period for each group [19]. Average cDDD per day was calculated as the sum of dispensed DDD divided by number of days exposed to study drugs within the exposure time period.

### Outcomes of Interest

After the 180-day drug exposure window, eligible patients were prospectively followed-up until the performance of second stone procedures (ESWL, URSL, ureterolithotomy, nephrolithotomy or PCNL), loss to follow-up, or the end of study date (December 31, 2010), whichever came first. The performance of a second stone procedure was our proxy for clinically significant stone recurrence.

To validate the accuracy of ESWL or URSL procedure in NHIRD, we randomly selected ~10% medical records from one medical center and one community teaching hospital in 2008. Nine hundred seventy-eight and 659 patients received procedures (ESWL, URSL, or both) in this medical center and community teaching hospital, respectively. We reviewed 98 out of 978 and 66 out of 659 medical records and found accuracy to be 100%.

### Nested Case-Control Study

A nested case-control study was also conducted with the same cohort. An incidence density sampling approach was used to match stone recurrence patients with controls (1:1) based on age, gender, and date of starting study drugs after 180-day complete treatment period to ensure the same drug exposure time window [18]. After matching, we assigned the date of stone recurrence in each case to his/her matched control at the end of follow-up. cDDD and average cDDD per day for each case and control within the matched drug exposure time period were also calculated [19].

### Potential Confounders

Several potential confounders that may affect the association between α-blocker drug use and the stone recurrence were considered in the analysis. Potential confunders included patients’ sociodemographic characteristics, patients’ index stone procedures, patients’ baseline comorbidities (S2 Table), use of double J tube, and the prescriptions of medications such as allopurinol, thiazide, potassium citrate [8] and antibiotics that were commonly used for the control of urinary tract infection in clinical practice, including cephalosporins, penicillins, fluoroquinolones, trimethoprim-sulfamethoxazole and nitrofurantoin.

### Statistical Analyses

Patients’ demographics and clinical characteristics were analyzed by percentage of total number of days of study drugs use within 180-day drug exposure window by quartile (≤ 25%, >25% and ≤ 50%, >50% and ≤ 75%, and >75%). Cox proportional hazard models were used to compute hazard ratios (HRs) with accompanying 95% confidence interval (CI). To meet the proportional hazards assumption, all dichotomous variables in the model were checked for proportionality using diagnostic log-log survival plots.

To ensure the robustness of results, the percentage of total number of days of study drugs use within 180-day drug exposure window was also divided into three groups: ≤ 10%, >10% and ≤ 80%, and >80%. A forest plot was used for sensitivity analysis for patients overall and patients with or without HTN and/or BPH comorbidities.

For the nested case-control study, conditional logistic regression models were used to examine the effect of drug exposure among all patients with recurring cases and their matched controls. The study drugs were analyzed by percentage of total number of days of study drugs use within 180-day drug exposure window by quartile, as well as by cDDD and average cDDD per day in the matched drug exposure time period by quartile. In addition to analyzing by the 180-day drug exposure window, we also analyzed the robustness of our results using 90- and 270-day drug exposure windows. All statistical operations perform using STATA, version S.E. 11.2 (Stata Corp.). A two-tailed P value less than 0.05 was considered significant.

## Results

This study analyzed data from 1,259 patients, most of whom were male and aged between 40–64 years (Table 1). At index date, about sixty-six percent (66.2%) of the patients received ESWL only, 32.3% received URSL only, and a small proportion (1.6%) underwent both procedures.

**Table 1:** Demographics and Clinical Characteristics of Study Patients Categorized by Quartile of Percentage of Total Number of Day of Study Drugs Use Within 180-day Drug Exposure Window.

	All patients	Quartile 1	Quartile 2	Quartile 3	Quartile 4	
N	1,259	795	217	116	131	P-value^1^
			Mean ± SD			
Age at index date (yrs)	52.8 ± 12.3	50.8 ± 12.5	55.0 ± 11.3	55.8 ± 10.3	58.5 ± 11.8	<0.0001
Total cDDDs of study α-blocker drugs	29.58±37.57	10.16±10.41	40.19±20.99	64.06±31.73	99.34±54.02	<0.0001
Daily cDDDs of study α-blocker drugs	0.16±0.21	0.06±0.06	0.22±0.12	0.36±0.18	0.55±0.30	<0.0001
Age (yrs)			N (%)			
<40	182 (14.5%)	150 (18.9%)	18 (8.3%)	6 (5.2%)	8 (6.1%)	<0.0001
40–64	853 (67.8%)	527 (66.3%)	156 (71.9%)	85 (73.3%)	85 (64.9%)	0.204
≥65	224 (17.8%)	118 (14.8%)	43 (19.8%)	25 (21.6%)	38 (29.0%)	0.001
Gender						
Male	1,082 (85.9%)	672 (84.5%)	196 (90.3%)	107 (92.2%)	107 (81.7%)	0.014
Female	177 (14.1%)	123 (15.5%)	21 (9.7%)	9 (7.8%)	24 (18.3%)	0.014
Geographic region						
Northern	601 (47.7%)	372 (46.8%)	106 (48.9%)	60 (51.7%)	63 (48.1%)	0.768
Central	338 (26.9%)	214 (26.9%)	56 (25.8%)	34 (29.3%)	34 (26.0%)	0.912
Eastern	280 (22.2%)	186 (23.4%)	45 (20.7%)	19 (16.4%)	30 (22.9%)	0.357
Southern	40 (3.2%)	23 (2.9%)	10 (4.6%)	3 (2.6%)	4 (3.1%)	0.617
Urbanization level						
Rural area	134 (10.6%)	85 (10.7%)	25 (11.5%)	9 (7.8%)	15 (11.5%)	0.733
Satellite city	432 (34.3%)	283 (35.6%)	65 (30.0%)	39 (33.6%)	45 (34.4%)	0.487
Urban	693 (55.0%)	427 (53.7%)	127 (58.5%)	68 (58.6%)	71 (54.2%)	0.518
Income (NTD per month, $)						
No Income or dependents	407 (32.3%)	225 (28.3%)	79 (36.4%)	44 (37.9%)	59 (45.0%)	<0.001
1–19,999	453 (36.0%)	287 (36.1%)	82 (37.8%)	43 (37.1%)	41 (31.3%)	0.655
20,000–39,999	276 (21.9%)	193 (24.3%)	40 (18.4%)	19 (16.4%)	24 (18.3%)	0.066
≥40,000	123 (9.8%)	90 (11.3%)	16 (7.4%)	10 (8.6%)	7 (5.3%)	0.083
Index stone procedure						
ESWL only	833 (66.2%)	517 (65.0%)	148 (68.2%)	78 (67.2%)	90 (68.7%)	0.730
URSL only	406 (32.3%)	262 (33.0%)	68 (31.3%)	37 (31.9%)	39 (29.8%)	0.887
Both ESWL and URSL	20 (1.6%)	16 (2.0%)	1 (0.5%)	1 (0.9%)	2 (1.5%)	0.380
Index stone procedure season						
January-March	240 (19.1%)	138 (17.4%)	49 (22.6%)	26 (22.4%)	27 (20.6%)	0.232
April-June	331 (26.3%)	201 (25.3%)	55 (25.4%)	36 (31.0%)	39 (29.8%)	0.443
July-September	408 (32.4%)	274 (34.5%)	65 (30.0%)	36 (31.0%)	33 (25.2%)	0.148
October-December	280 (22.2%)	182 (22.9%)	48 (22.1%)	18 (15.5%)	32 (24.4%)	0.309
Presence of second stone procedure	167 (13.3%)	112 (14.1%)	29 (13.4%)	16 (13.8%)	10 (7.6%)	0.250
Method of second stone procedure						
ESWL only	124 (9.9%)	82 (10.3%)	21 (9.7%)	11 (9.5%)	10 (7.6%)	0.815
URSL only	39 (3.1%)	28 (3.5%)	6 (2.8%)	5 (4.3%)	0	0.150
Both ESWL and ureterscopy	3 (0.2%)	2 (0.3%)	1 (0.5%)	0	0	0.790
Ureterolithotomy only	1 (0.1%)	0	1 (0.5%)	0	0	0.187
Presence of chronic diseases^2^						
Diabetes	177 (14.1%)	96 (12.1%)	35 (16.1%)	18 (15.5%)	28 (21.4%)	0.025
Hypertension	387 (30.7%)	197 (24.8%)	83 (38.3%)	41 (35.3%)	66 (50.4%)	<0.0001
Hyperlipidemia	187 (14.9%)	116 (14.6%)	36 (16.6%)	16 (13.8%)	19 (14.5%)	0.879
Gout	176 (14.0%)	107 (13.5%)	33 (15.2%)	20 (17.2%)	16 (12.2%)	0.611
Chronic kidney disease	28 (2.2%)	18 (2.3%)	4 (1.8%)	1 (0.9%)	5 (3.8%)	0.446
Osteoporosis	53 (4.2%)	30 (3.8%)	12 (5.5%)	4 (3.5%)	7 (5.3%)	0.594
BPH	69 (5.5%)	33 (4.2%)	15 (6.9%)	7 (6.0%)	14 (10.7%)	0.015
Types of study α-blocker drug use^3^						
Tamsulosin	712 (56.6%)	462 (58.1%)	125 (57.6%)	62 (53.5%)	63 (48.1%)	0.161
Terazosin	411 (32.6%)	219 (27.6%)	79 (36.4%)	46 (39.7%)	67 (51.2%)	<0.0001
Doxazosin	474 (37.7%)	234 (29.4%)	108 (49.8%)	62 (53.5%)	70 (53.4%)	<0.0001
Alfuzosin	177 (14.1%)	92 (11.6%)	34 (15.7%)	24 (20.7%)	27 (20.6%)	0.004
Other drugs use and treatment procedure^4^						
Allopurinol	89 (7.1%)	52 (6.5%)	16 (7.4%)	9 (7.8%)	12 (9.2%)	0.723
Citrate	54 (4.3%)	34 (4.3%)	8 (3.7%)	8 (6.9%)	4 (3.1%)	0.457
Thiazide	178 (14.1%)	98 (12.3%)	38 (17.5%)	15 (12.9%)	27 (20.6%)	0.031
Antibiotics^5^	934 (74.2%)	579 (72.8%)	161 (74.2%)	93 (80.2%)	101(77.1%)	0.319
Use of double J tube	8 (0.6%)	4 (0.5%)	1 (0.5%)	2 (1.7%)	1 (0.8%)	0.469

Abbreviation: BPH = Benign prostatic hyperplasia; cDDD = cumulative defined daily dose; ESWL = extracorporeal shock-wave lithotripsy; NTD = national Taiwan dollars; SD = standard deviation; PCNL = percutaneous nephrostolithotomy; URSL = ureterorenoscopic lithotripsy.

^1^P-value is to compare four study drug groups. ANOVA and Chi-square statistics were used for continuous variables and categorical variables, respectively.

^2^Statuses during one year before index date.

^3^Study drugs were used within 180-day study drug exposure window. These categories are not mutual exclusive.

^4^Other drugs and treatment procedures were used between index date+180 days and the end date of 180-day drug exposure window. These categories were not mutual exclusive.

^5^Antibiotics included cephalosporins, penicillins, fluoroquinolones, trimethoprim-sulfamethoxazole and nitrofurantoin.

**Fig 2:**
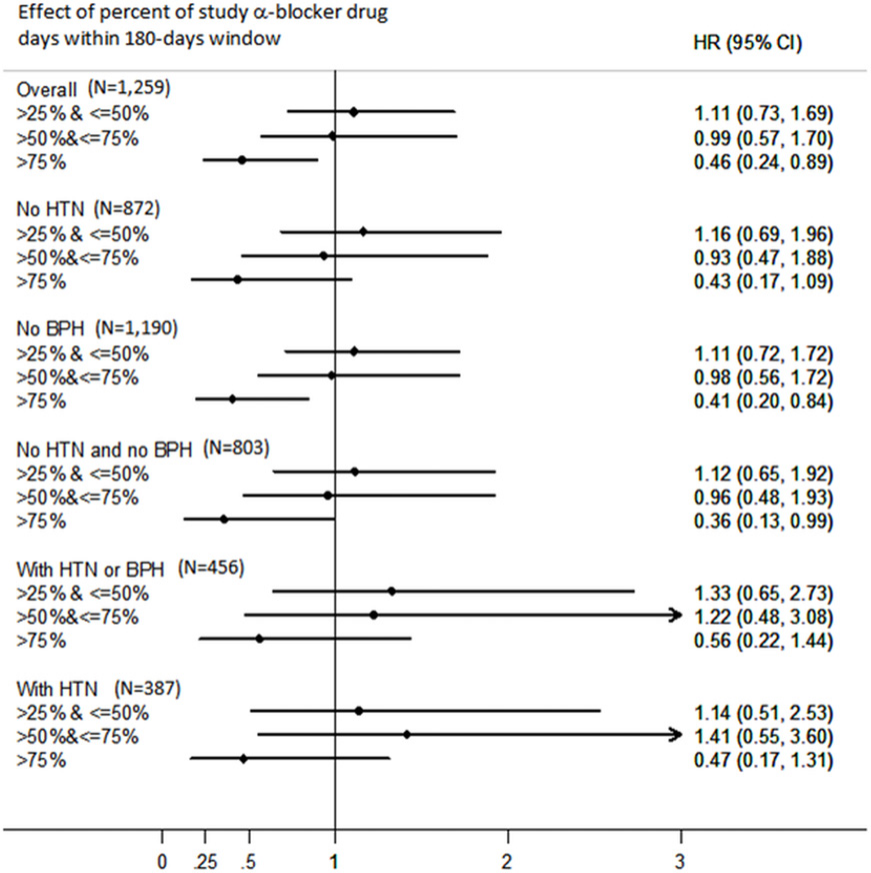
Sensitivity analysis of the effect of study α-blocker drug days within 180-days window on stone recurrence by hypertension (HTN) or/and benign prostate hypertrophy (BPH).

Urolithiasis was recurred and needed for surgical intervention in 167 patients during the 3,980 person-year follow-up, making an overall recurrence rate of 42 per 1,000 person-years. An inverse relationship was found between percent of days that the study drug was prescribed within 180-day drug exposure window and stone recurrence rates (S2 Fig). The recurrence rates were 45.64, 47.19, 43.11, and 18.52 per 1,000 person-years for patients in first to fourth percentage quartiles, respectively (Table 2). After adjusting for all covariates, the adjusted hazard ratios (HRs) were 1.11 (95% CI = 0.73 to 1.67) for the second quartile, 0.99 (95% CI = 0.57 to 1.70) for the third quartile, and 0.46 (95% CI = 0.24 to 0.89) for the fourth quartile, compared to the first quartile (Table 2 and S3 Table). The results were similar even after recategorizing the groups by different percentages of days prescribed (≤ 10%, >10% and ≤ 80%, and >80%) (Table 2). Even in patients categorized by HTN and/or BPH, there was a reduction in stone recurrence in the fourth quartile, significantly so in patients without BPH and without both BPH and HTN (Fig 2).

**Table 2:** Relationship of Percent of Daily Use of Study Drugs Within 180-day Drug Exposure Window with Recurrence of Urolithiasis in Cox Proportional Models.

Percentage of total number of days of study drugs use within 180-day drug exposure window	No. of patients	No. of person-years	No. of patients with recurrence	Recurrent rate (per 1,000 person-years)	Crude HR (95% CI)	P-value	Adjusted HR^1^ (95% CI)	P-value
By quartile								
Quartile 1	795	2,454	112	45.64	1.00		1.00	
Quartile 2	217	615	29	47.19	1.02 (0.68, 1.54)	0.911	1.11 (0.73,1.69)	0.637
Quartile 3	116	371	16	43.11	0.96 (0.57, 1.62)	0.870	0.99 (0.57,1.70)	0.960
Quartile 4	131	540	10	18.52	0.42 (0.22, 0.81)	0.009	0.46 (0.24,0.89)	0.022
Three groups								
≤10%	504	1,511	69	45.67	1.00		1.00	
>10% and ≤80%	639	1,975	90	45.57	1.01 (0.74, 1.38)	0.969	0.99 (0.72,1.38)	0.965
>80%	116	494	8	16.18	0.37 (0.18, 0.77)	0.008	0.41 (0.19,0.86)	0.018

Abbreviation: HR = Hazard ratio; CI = Confidence interval

^1^Adjusting for all variables listed in Table 1.

In the nested case-control study, the case and matched controls were similar in almost all variables, except history of gout and prescription of potassium citrate (S4 Table). Their average follow-up times were the same (mean ± SD = 602.0 ± 463.5 days). We performed a similar analysis in this case-control study, and found that, when using the 180-day drug exposure window, the adjusted ORs was 0.23 (95% CI = 0.10 to 0.53, P = 0.001) in the fourth quartile, compared to the first, after adjusting for all covariates except age and gender (Table 3). The results remained similar even in patients categorized by cDDD quartiles and average cDDD per day quartiles. In the sensitivity analysis, the results remained similar after changing drug exposure period from 180 days to either 90 days or 270 days (S5 Table).

**Table 3:** Relationship of Different Study Drugs Exposure with Recurrence of Urolithiasis in Conditional Logistic Regression Models.

	Cases	Controls	Crude OR		Adjusted OR^1^	
	N = 167	N = 167	(95% CI)	P-value	(95% CI)	P-value
	N (%)					
Percent of daily use of study drugs within 180-day drug exposure window by quartile^2^						
Quartile 1	112 (67.1)	93 (55.7)	1.00		1.00	
Quartile 2	29 (17.4)	22 (13.2)	1.10 (0.59, 2.03)	0.764	1.03 (0.52, 2.06)	0.926
Quartile 3	16 (9.6)	20 (12.0)	0.62 (0.27, 1.38)	0.239	0.41 (0.16, 1.08)	0.072
Quartile 4	10 (6.0)	32 (19.2)	0.30 (0.15, 0.63)	0.001	0.23 (0.10, 0.53)	0.001
Percent of daily use of study drugs within 180-day drug exposure window by 3 groups^2^						
≤10%	69 (41.3)	69 (41.3)	1.00		1.00	
>10% and ≤80%	90 (53.9)	71 (42.5)	1.24 (0.78, 1.97)	0.371	1.14 (0.67, 1.93)	0.634
>80%	8 (4.8)	27 (16.2)	0.34 (0.14, 0.77)	0.010	0.27 (0.10, 0.71)	0.007
Total cDDD use of study drugs within matched exposure time period by quartile^3^						
Quartile 1	48 (28.7)	36 (21.6)	1.00		1.00	
Quartile 2	40 (24.0)	42 (25.2)	0.81 (0.48, 1.38)	0.435	0.83 (0.45, 1.51)	0.534
Quartile 3	41 (24.6)	44 (26.4)	0.75 (0.42, 1.34)	0.329	0.81 (0.43, 1.53)	0.524
Quartile 4	38 (22.8)	45 (27.0)	0.67 (0.36, 1.21)	0.184	0.45 (0.22, 0.92)	0.029
Total cDDD use of study drugs by day within matched exposure time period by quartile^3^						
Quartile 1	47 (28.1)	36 (21.6)	1.00		1.00	
Quartile 2	45 (27.0)	39 (23.4)	1.00 (0.57, 1.75)	0.999	1.12 (0.59, 2.14)	0.734
Quartile 3	42 (25.2)	41 (24.6)	0.83 (0.47, 1.47)	0.521	0.82 (0.43, 1.56)	0.545
Quartile 4	33 (19.8)	51 (30.5)	0.51 (0.28, 0.92)	0.025	0.35 (0.17, 0.70)	0.003

Abbreviation: cDDDs = Cumulative defined daily dose.

^1^ Adjusting for all variables listed in Table 1 except age and gender.

^2^ A 180-day drug exposure window was defined as the use of study drug days within 180 days from the date of first pharmacy claim after 180-day complete treatment period.

^3^ As described in the method section, we matched case and control based on their age, gender and date of first pharmacy claim after the 180-day complete treatment period. After matching, we assigned the date of stone recurrence in each case to his/her matched control at the end of follow-up.

## Discussion

This study found that patients prescribed α-blockers more frequently in the180-day drug exposure window after the first stone episode had a significantly reduced risk of recurring stones requiring further surgical intervention, with the fourth quartile having an adjusted HR of 0.46 (95% CI = 0.24 to 0.89) versus the first quartile (Table 2). These results remained significant in the nested case-control study and even when recategorizing the patient groups cDDD and average cDDD per day and in the sensitivity study when the number of days of the exposure was changed.

Because of the advances in the understanding of ureteral smooth-muscle physiology and obstruction caused by urinary stones, it has been suggested that α-blockers can facilitate urolithiasis expulsion due to decrease the force and frequency of ureteric contractions and increase the fluid bolus volume transported the ureter [20–22]. Several smaller clinical trials have reported that short-term use of α-blockers in less than 90 days can facilitate the passage of ureteral stones with and without surgical intervention. These findings were also confirmed in meta-analyses reviewing clinical trial studies [9–10]. One recent meta-analysis pooling 47 randomized and controlled trials by Seitz *et al*. provided the evidence that users of α-blocker had a higher and faster expulsion rate of ureteral stones compared to controls (RR = 1.45; 95% CI = 1.34 to 1.57) [9]. Although there is a great need for meta-analysis, one should be cautious that they are likely affected by publication bias generating positive and significant findings [10].

In addition, Karabacak et al has confirmed the presence and distribution of α1 receptors and subtypes in human pelvis and calyces recently [11]. Their findings imply that α-blocker treatment could facilitate kidney stone passage and help decrease pain, as used in ureteral stones [11]. Several updated randomized clinical trials also confirmed the adjuvant effect of α-blockers on improving stone free rates after surgical intervention of renal stones [23–25]. All studies consistently suggested the beneficial effect of α-blocker on stone recurrence; the possible mechanism may be due to the prevention of stone retention in the kidney and facilitation of stone passage via ureter. However, these studies are also limited by small sample size (a few hundred study patients) and a short periods of follow-up time (from several weeks up to less than three months). The findings from the current large-scale, relatively long-term follow-up study fill an important gap in research knowledge and provide solid evidence of the protective effect of α-blocker on recurrence of urolithiasis needed for surgical intervention.

α-blockers are widely used in the treatment of HTN [26] and BPH [27]. To avoid the potential confounding effect by disease entity of HTN and/or BPH, we also analyzed our results by these variables and found the study drugs to have a protective effect against stone recurrence in the group exposed to the highest percent of daily study drug use, compared to the group exposed lowest percent of days, within the 180-day drug exposure window. These findings suggest that the protective effect of α-blockers use on recurrent stones needed for surgical intervention was not affected by different comorbidity of chronic diseases. Unexpectedly, patients prescribed potassium citrate in this study seemed to be at increased risk of recurrence. One possible explanation for this increased risk might be related to confounding by indication, as potassium citrate is generally considered a relatively safe and commonly used medication in the prevention of stone recurrence in patients at potentially high risk of developing urolithiasis by physicians [8].

This study has several limitations. One limitation is that, the exposure of interest was prescription information of α-blockers obtained from the NHI research dataset. We cannot know whether the patients adhered to the prescribed regimen, a bias that might cause random misclassification of exposure interest and underestimates in our findings. Another limitation is that the database we used did not provide data on several important lifestyle factors such as daily intake of fluid amount or obesity [28] and stone composition. Our use of these procedure as proxy for recurrence also meant that the recurrence of small stones not requiring surgery may have been missed, possibly leading to underestimation of recurrence and limiting our findings to more severe cases. Despite these limitations, given that most clinical trials are limited to small samples and short periods of follow-up, there has been a need for a large and high-quality confirmatory trial to confirm the benefit of medical treatment, especially use of α-blocker, on urinary stone passage or recurrence [10]. This current study is a nationwide population-based study with both cohort and case-control design with sufficient sample size testing different time exposure windows.

## Conclusions

This study found that use of α-blocker for 180 days or more prevent recurrence of urolithiasis needed for surgical intervention. In patients at higher risk of recurrent urolithiasis, long term prescription of α-blockers might help them prevent further surgical intervention. Further large prospective studies are needed to confirm our preliminary results.

## Supporting Information

S1 Fig Diagram of study design. (TIF)

S2 Fig Association between predicted hazard ratio and percentage of days of α-blockers drug use within a 180-day drug exposure window. (TIF)

S1 Table ICD-9-CM procedures and NHI billing codes for procedure of stone removal. (DOCX)

S2 Table ICD-9-CM diagnosis codes for comorbidities. (DOCX)

S3 Table Defined daily dose of study α-blockers. (DOCX)

S4 Table Variables associated with Recurrence of urolithiasis in Cox proportional models. (DOCX)

S5 Table Demographics and clinical characteristics among all recurrent cases and their matched controls. (DOCX)

S6 Table Relationship of α-blocker use and recurrence of urolithiasis within 90- and 270-day study drug exposure windows in conditional logistic regression models. (DOCX)
