# Supplementary figures and images for: Long-Term Prescription of α-Blockers Decrease the Risk of Recurrent Urolithiasis Needed for Surgical Intervention-A Nationwide Population-Based Study
Source: PLoS One. 2015 Apr 13;10(4):e0122494. doi: 10.1371/journal.pone.0122494 (PMC4395263; doi:10.1371/journal.pone.0122494)

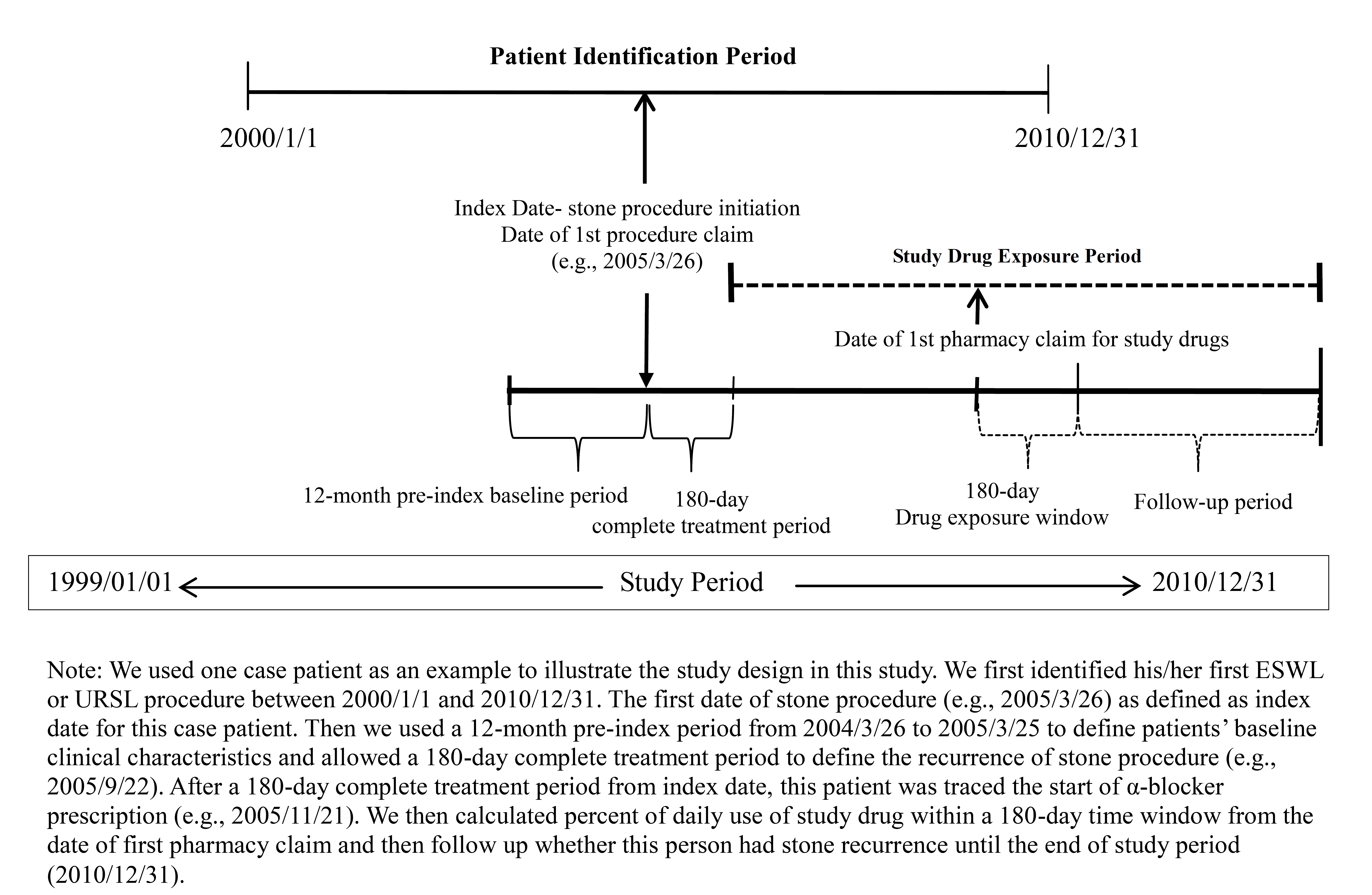

Supplement: S1 Fig — (TIF) [file pone.0122494.s001.tif]

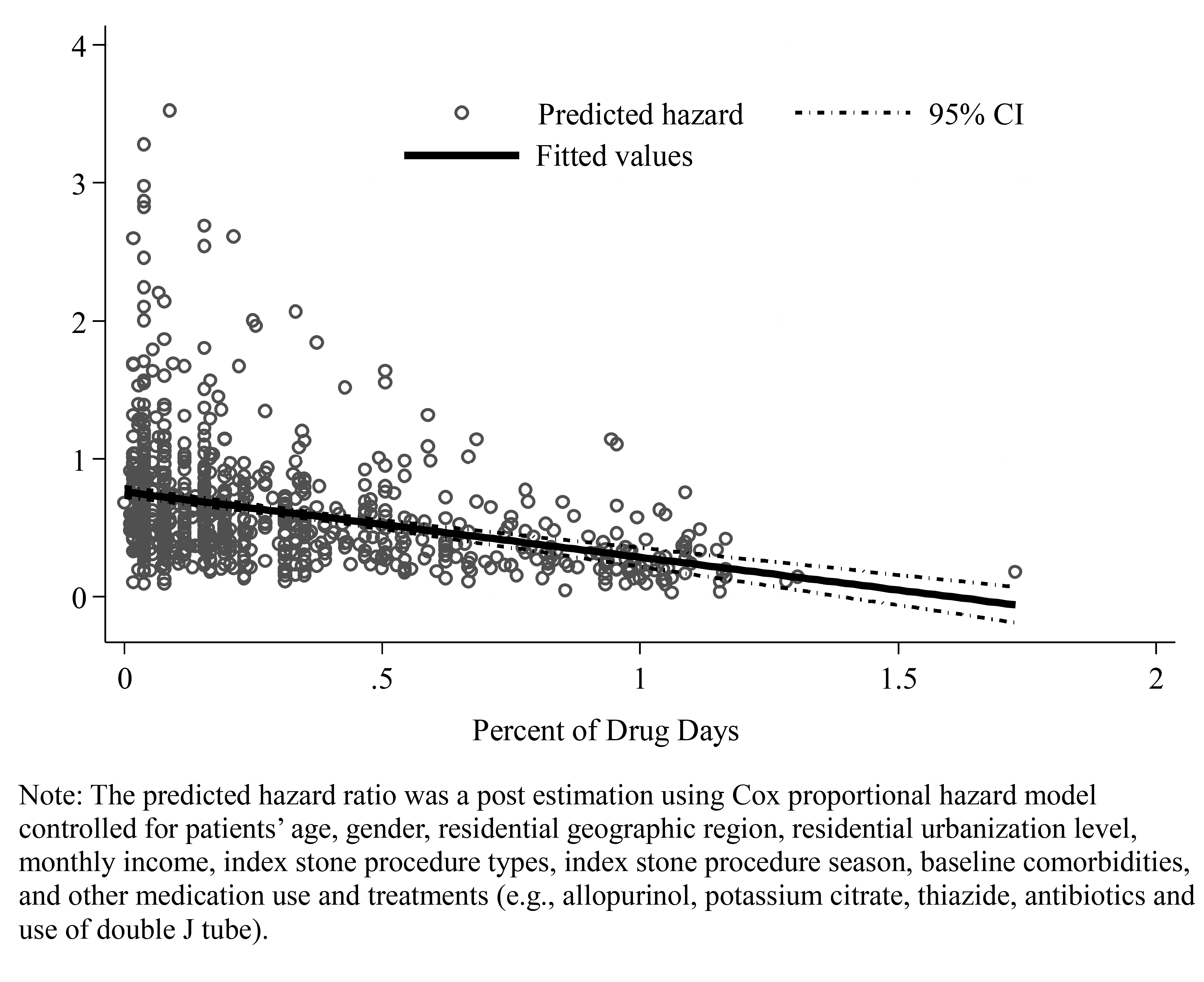

Supplement: S2 Fig — (TIF) [file pone.0122494.s002.tif]
