# Supplementary material for: Long-Term Prescription of α-Blockers Decrease the Risk of Recurrent Urolithiasis Needed for Surgical Intervention-A Nationwide Population-Based Study
Source: PLoS One. 2015 Apr 13;10(4):e0122494. doi: 10.1371/journal.pone.0122494 (PMC4395263; doi:10.1371/journal.pone.0122494)
Supplement: S1 Table — (DOCX) [file pone.0122494.s003.docx]

## S1 Table. ICD-9-CM procedures and NHI billing codes for procedure of stone removal.

| **Stone Procedure** | **NHI Order Code/ Treatment Billing Codes** | **ICD Procedure Codes** |
| --- | --- | --- |
| ESWL | 50023A, 50023B, 50024A, 50024B | 98.51 |
| URSL | 77026B, 77027B, 77028B |  |
| Ureterolithotomy | 77001B, 77002B, 77030B |  |
| Percutaneous nephrostolithotomy (PCNL) | 76016B, 76017B |  |
| Nephrolithotomy | 76011B, 76012B, 76023B, 77032B |  |
