# Supplementary material for: Long-Term Prescription of α-Blockers Decrease the Risk of Recurrent Urolithiasis Needed for Surgical Intervention-A Nationwide Population-Based Study
Source: PLoS One. 2015 Apr 13;10(4):e0122494. doi: 10.1371/journal.pone.0122494 (PMC4395263; doi:10.1371/journal.pone.0122494)
Supplement: S2 Table — (DOCX) [file pone.0122494.s004.docx]

## S2 Table. ICD-9-CM diagnosis codes for comorbidities.

| **Diseases** | **ICD-9-CM Codes** | **A Codes** |
| --- | --- | --- |
| Diabetes mellitus | 250.xx | A181 |
| Hypertension | 401.xx, 402.xx, 403.xx, 404.xx | A260, A269 |
| Hyperlipidemia | 272.xx |  |
| Gout | 274.xx |  |
| Coronary heart disease | 410.xx-414.xx, 429.2 | A279 |
| Chronic kidney disease | 585.xx | A350 |
| Osteoporosis | 733.xx | A439 |
| Benign prostatic hyperplasia (BPH) | 600.xx |  |
