# Supplementary material for: Long-Term Prescription of α-Blockers Decrease the Risk of Recurrent Urolithiasis Needed for Surgical Intervention-A Nationwide Population-Based Study
Source: PLoS One. 2015 Apr 13;10(4):e0122494. doi: 10.1371/journal.pone.0122494 (PMC4395263; doi:10.1371/journal.pone.0122494)
Supplement: S3 Table — (DOCX) [file pone.0122494.s005.docx]

**S3 Table. Defined daily dose of study α-blockers.**

| **α-blockers** | **DDD ( Defined daily dose)** |
| --- | --- |
| Tamsulosin | 0.4 mg |
| Terazosin | 5 mg |
| Doxazosin | 4 mg |
| Alfuzosin | 7.5 mg |
