# Supplementary material for: Long-Term Prescription of α-Blockers Decrease the Risk of Recurrent Urolithiasis Needed for Surgical Intervention-A Nationwide Population-Based Study
Source: PLoS One. 2015 Apr 13;10(4):e0122494. doi: 10.1371/journal.pone.0122494 (PMC4395263; doi:10.1371/journal.pone.0122494)
Supplement: S4 Table — (DOCX) [file pone.0122494.s006.docx]

**S4 Table. Variables associated with Recurrence of urolithiasis in Cox proportional models.**

| Variables | No. of patients | No. of person-years | No. of patients with recurrence | Recurrent rate (per 1,000 person-years) | Crude HR  (95% CI) | P-value | Model 1^1^ | | Model 2^1^ | |
| --- | --- | --- | --- | --- | --- | --- | --- | --- | --- | --- |
|  |  |  |  |  |  |  | Adjusted HR  (95% CI) | P-value | Adjusted HR  (95% CI) | P-value |
| All patients | 1,259 | 3,980 | 167 | 41.96 |  |  |  |  |  |  |
|  |  |  |  |  |  |  |  |  |  |  |
| Percentage of total number of days of study drugs use within a 180-day drug exposure window by quartile |  |  |  |  |  |  |  |  |  |  |
| Quartile 1 | 795 | 2,454 | 112 | 45.64 | 1.00 |  | 1.00 |  | 1.00 |  |
|  |  |  |  |  |  |  |  |  |  |  |
| Quartile 2 | 217 | 615 | 29 | 47.19 | 1.02 | 0.911 | 1.08 | 0.724 | 1.11 | 0.637 |
|  |  |  |  |  | (0.68, 1.54) |  | (0.71,1.63) |  | (0.73,1.69) |  |
| Quartile 3 | 116 | 371 | 16 | 43.11 | 0.96 | 0.870 | 0.99 | 0.971 | 0.99 | 0.960 |
|  |  |  |  |  | (0.57, 1.62) |  | (0.58,1.69) |  | (0.57,1.70) |  |
| Quartile 4 | 131 | 540 | 10 | 18.52 | 0.42 | 0.009 | 0.46 | 0.02 | 0.46 | 0.022 |
|  |  |  |  |  | (0.22, 0.81) |  | (0.24,0.88) |  | (0.24,0.89) |  |
| Age (yrs) |  |  |  |  |  |  |  |  |  |  |
| < 40 | 182 | 446 | 28 | 62.72 | 1.00 |  | 1.00 |  | 1.00 |  |
|  |  |  |  |  |  |  |  |  |  |  |
| 40-64 | 853 | 2,658 | 110 | 41.39 | 0.69 | 0.076 | 0.78 | 0.254 | 0.77 | 0.255 |
|  |  |  |  |  | (0.45,1.04) |  | (0.50,1.20) |  | (0.49,1.21) |  |
| ≥ 65 | 224 | 875 | 29 | 33.13 | 0.57 | 0.035 | 0.74 | 0.308 | 0.70 | 0.234 |
|  |  |  |  |  | (0.34,0.96) |  | (0.42,1.32) |  | (0.38,1.26) |  |
| Gender |  |  |  |  |  |  |  |  |  |  |
| Male | 1,082 | 3,409 | 141 | 41.36 | 1.00 |  | 1.00 |  | 1.00 |  |
|  |  |  |  |  |  |  |  |  |  |  |
| Female | 177 | 570 | 26 | 45.58 | 1.10 | 0.666 | 1.32 | 0.247 | 1.40 | 0.169 |
|  |  |  |  |  | (0.72,1.67) |  | (0.83,2.10) |  | (0.87,2.25) |  |
| Geographical region |  |  |  |  |  |  |  |  |  |  |
| Northern | 601 | 1,889 | 85 | 45.00 | 1.00 |  |  |  | 1.00 |  |
|  |  |  |  |  |  |  |  |  |  |  |
| Central | 338 | 1,078 | 38 | 35.26 | 0.79 | 0.225 |  |  | 0.72 | 0.120 |
|  |  |  |  |  | (0.54,1.16) |  |  |  | (0.47,1.09) |  |
| Eastern | 280 | 895 | 39 | 43.56 | 0.98 | 0.902 |  |  | 0.94 | 0.777 |
|  |  |  |  |  | (0.67,1.43) |  |  |  | (0.63,1.41) |  |
| Southern | 40 | 118 | 5 | 42.50 | 0.92 | 0.850 |  |  | 0.88 | 0.794 |
|  |  |  |  |  | (0.37,2.26) |  |  |  | (0.34,2.26) |  |
| Urbanization |  |  |  |  |  |  |  |  |  |  |
| Rural area | 134 | 409 | 14 | 34.19 | 1.00 |  |  |  | 1.00 |  |
|  |  |  |  |  |  |  |  |  |  |  |
| Satellite city | 432 | 1,453 | 67 | 46.10 | 1.39 | 0.263 |  |  | 1.25 | 0.476 |
|  |  |  |  |  | (0.78,2.47) |  |  |  | (0.68,2.28) |  |
| Urban | 693 | 2,117 | 86 | 40.63 | 1.18 | 0.557 |  |  | 0.96 | 0.903 |
|  |  |  |  |  | (0.67,2.08) |  |  |  | (0.51,1.80) |  |
| Income (Taiwan dollars per month) |  |  |  |  |  |  |  |  |  |  |
| No or dependent | 407 | 1,459 | 53 | 36.33 | 1.00 |  |  |  | 1.00 |  |
|  |  |  |  |  |  |  |  |  |  |  |
| $1- 19,999 | 453 | 1,385 | 61 | 44.04 | 1.18 | 0.383 |  |  | 1.00 | 0.990 |
|  |  |  |  |  | (0.81,1.70) |  |  |  | (0.68,1.48) |  |
| $20,000- $39,999 | 276 | 784 | 31 | 39.55 | 1.05 | 0.825 |  |  | 0.80 | 0.359 |
|  |  |  |  |  | (0.67,1.64) |  |  |  | (0.50,1.29) |  |
| ≥$40000 | 123 | 352 | 22 | 62.55 | 1.66 | 0.047 |  |  | 1.37 | 0.247 |
|  |  |  |  |  | (1.01,2.73) |  |  |  | (0.80,2.33) |  |
| Index stone procedure |  |  |  |  |  |  |  |  |  |  |
| ESWL only | 833 | 2,632 | 117 | 44.45 | 1.00 |  | 1.00 |  | 1.00 |  |
|  |  |  |  |  |  |  |  |  |  |  |
| URSL only | 406 | 1,299 | 46 | 35.42 | 0.80 | 0.188 | 0.74 | 0.096 | 0.79 | 0.181 |
|  |  |  |  |  | (0.57,1.12) |  | (0.53,1.05) |  | (0.55,1.12) |  |
| Both ESWL and URSL | 20 | 49 | 4 | 82.33 | 1.72 | 0.286 | 2.27 | 0.112 | 2.47 | 0.082 |
|  |  |  |  |  | (0.63,4.67) |  | (0.83,6.25) |  | (0.89,6.88) |  |
| Season for indexed stone procedure |  |  |  |  |  |  |  |  |  |  |
| January-March | 240 | 830 | 33 | 39.76 | 1.00 |  |  |  | 1.00 |  |
|  |  |  |  |  |  |  |  |  |  |  |
| April-June | 331 | 1,054 | 42 | 39.83 | 0.99 | 0.955 |  |  | 0.99 | 0.949 |
|  |  |  |  |  | (0.63,1.56) |  |  |  | (0.62,1.57) |  |
| July-September | 408 | 1,213 | 62 | 51.10 | 1.24 | 0.312 |  |  | 1.27 | 0.274 |
|  |  |  |  |  | (0.81,1.90) |  |  |  | (0.83,1.97) |  |
| October-December | 280 | 882 | 30 | 34.03 | 0.84 | 0.477 |  |  | 0.86 | 0.554 |
|  |  |  |  |  | (0.51,1.37) |  |  |  | (0.52,1.42) |  |
| Medical diseases^2^ |  |  |  |  |  |  |  |  |  |  |
| Diabetes |  |  |  |  |  |  |  |  |  |  |
| No | 1,082 | 3,332 | 149 | 44.72 | 1.00 |  | 1.00 |  | 1.00 |  |
| Yes | 177 | 648 | 18 | 27.80 | 0.64 | 0.077 | 0.73 | 0.230 | 0.73 | 0.238 |
|  |  |  |  |  | (0.39,1.05) |  | (0.44,1.22) |  | (0.44,1.23) |  |
| Hypertension |  |  |  |  |  |  |  |  |  |  |
| No | 872 | 2,620 | 120 | 45.80 | 1.00 |  | 1.00 |  | 1.00 |  |
| Yes | 387 | 1,359 | 47 | 34.57 | 0.77 | 0.135 | 0.92 | 0.687 | 0.95 | 0.807 |
|  |  |  |  |  | (0.55,1.08) |  | (0.63,1.36) |  | (0.64,1.41) |  |
| Hyperlipidemia |  |  |  |  |  |  |  |  |  |  |
| No | 1,072 | 3,357 | 146 | 43.49 | 1.00 |  | 1.00 |  | 1.00 |  |
| Yes | 187 | 622 | 21 | 33.75 | 0.79 | 0.306 | 0.75 | 0.258 | 0.73 | 0.229 |
|  |  |  |  |  | (0.50,1.24) |  | (0.45,1.24) |  | (0.44,1.21) |  |
| Gout |  |  |  |  |  |  |  |  |  |  |
| No | 1,083 | 3,413 | 135 | 39.55 | 1.00 |  | 1.00 |  | 1.00 |  |
| Yes | 176 | 566 | 32 | 56.51 | 1.43 | 0.070 | 1.97 | 0.002 | 2.00 | 0.002 |
|  |  |  |  |  | (0.97,2.10) |  | (1.28,3.03) |  | (1.29,3.10) |  |
| Chronic kidney disease |  |  |  |  |  |  |  |  |  |  |
| No | 1,231 | 3,853 | 165 | 42.82 | 1.00 |  | 1.00 |  | 1.00 |  |
| Yes | 28 | 126 | 2 | 15.84 | 0.39 | 0.185 | 0.38 | 0.182 | 0.38 | 0.185 |
|  |  |  |  |  | (0.10,1.57) |  | (0.09,1.57) |  | (0.09,1.59) |  |
| Osteoporosis |  |  |  |  |  |  |  |  |  |  |
| No | 1,206 | 3,760 | 155 | 41.22 | 1.00 |  | 1.00 |  | 1.00 |  |
| Yes | 53 | 219 | 12 | 54.75 | 1.40 | 0.266 | 1.57 | 0.155 | 1.60 | 0.142 |
|  |  |  |  |  | (0.77,2.52) |  | (0.84,2.91) |  | (0.85,2.98) |  |
| BPH |  |  |  |  |  |  |  |  |  |  |
| No | 1,190 | 3,689 | 159 | 43.10 | 1.00 |  | 1.00 |  | 1.00 |  |
| Yes | 69 | 291 | 8 | 27.51 | 0.67 | 0.266 | 0.77 | 0.495 | 0.78 | 0.522 |
|  |  |  |  |  | (0.33,1.36) |  | (0.37,1.62) |  | (0.37,1.65) |  |
| Other drugs and treatment uses^3^ |  |  |  |  |  |  |  |  |  |  |
| Allopurinol |  |  |  |  |  |  |  |  |  |  |
| No | 1,164 | 3,603 | 156 | 43.30 | 1.00 |  | 1.00 |  | 1.00 |  |
| Yes | 95 | 376 | 11 | 29.22 | 0.68 | 0.233 | 0.57 | 0.104 | 0.55 | 0.089 |
|  |  |  |  |  | (0.36,1.29) |  | (0.29,1.12) |  | (0.28,1.09) |  |
| Potassium citrate |  |  |  |  |  |  |  |  |  |  |
| No | 1,198 | 3,817 | 152 | 39.83 | 1.00 |  | 1.00 |  | 1.00 |  |
| Yes | 61 | 163 | 15 | 92.12 | 1.97 | 0.025 | 1.73 | 0.078 | 1.92 | 0.040 |
|  |  |  |  |  | (1.09,3.54) |  | (0.94,3.18) |  | (1.03,3.56) |  |
| Thiazide |  |  |  |  |  |  |  |  |  |  |
| No | 1,065 | 3,245 | 140 | 43.15 | 1.00 |  | 1.00 |  | 1.00 |  |
| Yes | 194 | 735 | 27 | 36.75 | 0.78 | 0.292 | 0.82 | 0.396 | 0.84 | 0.485 |
|  |  |  |  |  | (0.50,1.23) |  | (0.51,1.31) |  | (0.52,1.36) |  |
| Antibiotics |  |  |  |  |  |  |  |  |  |  |
| No | 325 | 1,112 | 54 | 48.57 |  |  |  |  |  |  |
| Yes | 934 | 2,868 | 113 | 39.41 | 0.79 | 0.151 |  |  | 0.72 | 0.053 |
|  |  |  |  |  | (0.57,1.09) |  |  |  | (0.51,1.00) |  |
| Use of double J tube |  |  |  |  |  |  |  |  |  |  |
| No | 1,251 | 3,961 | 164 | 41.41 |  |  |  |  |  |  |
| Yes | 8 | 19 | 3 | 158.23 | 3.60 | 0.028 | 3.38 | 0.041 | 3.30 | 0.050 |
|  |  |  |  |  | (1.15,11.27) |  | (1.05,10.89) |  | (1.00,10.88) |  |
| Abbreviation: HR=Hazard ratio; CI = Confidence interval; BPH = Benign prostatic hyperplasia; ESWL = Extracorporeal shock-wave lithotripsy; URSL = Ureterorenoscopic lithotripsy; PCNL = Percutaneous nephrostolithotomy.  ^1^Model 1 included patient clinical characteristics variables (e.g., age, gender, index procedure, percent of study drug use days, comorbidities, dummy variables for identifying other drug use and use of double J tube) in the cox model, whereas Model 2 included all the listed variables.  ^2^Statuses during one year before index date.  ^3^Other drugs and treatment procedures were used between index date+180 days and the end date of 180-day drug exposure window. | | | | | | | | | | |
