# Supplementary material for: Long-Term Prescription of α-Blockers Decrease the Risk of Recurrent Urolithiasis Needed for Surgical Intervention-A Nationwide Population-Based Study
Source: PLoS One. 2015 Apr 13;10(4):e0122494. doi: 10.1371/journal.pone.0122494 (PMC4395263; doi:10.1371/journal.pone.0122494)
Supplement: S5 Table — (DOCX) [file pone.0122494.s007.docx]

## S5 Table. Demographics and clinical characteristics among all recurrent cases and their matched controls.

|  | Cases | Controls | P-Value^1^ |
| --- | --- | --- | --- |
| N | 167 | 167 |  |
|  | N (%) or mean ± SD | |  |
| Age (yrs) |  |  |  |
| <40 | 28 (16.8) | 28 (16.8) | - |
| 40-64 | 110 (65.9) | 110 (65.9) |  |
| 65+ | 29 (17.4) | 29 (17.4) |  |
| Gender |  |  |  |
| Male | 141 (84.4) | 141 (84.4) | - |
| Female | 26 (15.6) | 26 (15.6) |  |
| Indexed Stone Procedure |  |  |  |
| ESWL only | 117 (70.1) | 122 (73.1) | 0.564 |
| URSL only | 46 (27.5) | 43 (25.8) | 0.729 |
| Both ESWL and URSL | 4 (2.4) | 2 (1.2) | 0.341 |
| Medical diseases^2^ |  |  |  |
| Diabetes | 18 (10.8) | 23 (13.8) | 0.356 |
| Hypertension | 47 (28.1) | 53 (31.7) | 0.439 |
| Hyperlipidemia | 21 (12.6) | 20 (12.0) | 0.869 |
| Gout | 32 (19.2) | 14 (8.4) | 0.007 |
| Chronic Kidney Disease | 2 (1.2) | 1 (0.6) | 0.571 |
| Osteoporosis | 12 (7.2) | 9 (5.4) | 0.469 |
| BPH | 8 (4.8) | 14 (8.4) | 0.207 |
| Matched drug exposure time period (days) | 601.96 ± 463.54 | 601.96 ± 463.54 | - |
| Use of study α-blocker drug within matched exposure time period^3^ |  |  |  |
| Tamsulosin | 74 (44.3) | 61 (36.5) | 0.165 |
| Terazosin | 51 (30.5) | 53 (31.7) | 0.803 |
| Doxazosin | 40 (24.0) | 56 (33.5) | 0.048 |
| Alfuzosin | 20 (12.0) | 14 (8.4) | 0.292 |
| Mean of total cDDDs of study α-blocker drug within matched exposure time period | 59.11 ± 119.47 | 67.40 ± 114.24 | 0.478 |
| Mean of average cDDDs per day of study α-blocker drug within matched exposure time period | 0.10 ± 0.17 | 0.14 ± 0.22 | 0.068 |
| Use of other drugs within matched exposure time period^3^ |  |  |  |
| Allopurinol | 1 (0.6) | 7 (4.2) | 0.069 |
| Potassium citrate | 11 (6.6) | 3 (1.8) | 0.046 |
| Thiazide | 19 (11.4) | 20 (12.0) | 0.866 |
| Antibiotics | 113 (67.7%) | 117 (70.1%) | 0.628 |
| Use of Double J tube | 3 (1.8%) | (0.0%) | 1.000 |
| Abbreviation: SD = Standard deviation; BPH = Benign prostatic hyperplasia; ESWL = Extracorporeal shock-wave lithotripsy; URSL = Ureterorenoscopic lithotripsy.  ^1^Conditional logisic regression was used for categorical variable and paired T-test was used for continuous variables.  ^2^Statuses during one year before index date.  ^3^ Other drugs and treatment procedures were used between index date+180 days and the end date of 180-day drug exposure window. These categories are not mutual exclusive. | | | |
