# Supplementary material for: Long-Term Prescription of α-Blockers Decrease the Risk of Recurrent Urolithiasis Needed for Surgical Intervention-A Nationwide Population-Based Study
Source: PLoS One. 2015 Apr 13;10(4):e0122494. doi: 10.1371/journal.pone.0122494 (PMC4395263; doi:10.1371/journal.pone.0122494)
Supplement: S6 Table — (DOCX) [file pone.0122494.s008.docx]

**S6 Table. Relationship of α-blocker use and recurrence of urolithiasis within 90- and 270-day study drug exposure windows in conditional logistic regression models.**

| Percentage of total number of days of study drugs use by quartile | Cases | Controls | Crude OR | | Adjusted OR^1^ | |
| --- | --- | --- | --- | --- | --- | --- |
|  | N (%) | | 95% CI | P-value | 95% CI | P-value |
| Within a 90-day drug exposure window^2^ | | | | | | |
|  | N = 194 | N = 194 |  |  |  |  |
| Quartile 1 | 96 (49.5) | 71 (36.6) | 1.00 |  | 1.00 |  |
|  |  |  |  |  |  |  |
| Quartile 2 | 46 (23.7) | 54 (27.8) | 0.64 | 0.086 | 0.64 | 0.119 |
|  |  |  | (0.38, 1.07) |  | (0.37, 1.12) |  |
| Quartile 3 | 23 (11.9) | 25 (12.9) | 0.63 | 0.193 | 0.55 | 0.104 |
|  |  |  | (0.32, 1.26) |  | (0.26, 1.13) |  |
| Quartile 4 | 29 (15.0) | 44 (22.7) | 0.44 | 0.010 | 0.41 | 0.011 |
|  |  |  | (0.23, 0.82) |  | (0.20, 0.81) |  |
| Within a 270-day drug exposure window^2^ | | | | | | |
|  | N = 149 | N = 149 |  |  |  |  |
| Quartile 1 | 112 (75.2) | 96 (64.4) | 1.00 |  | 1.00 |  |
|  |  |  |  |  |  |  |
| Quartile 2 | 20 (13.4) | 19 (12.8) | 0.92 | 0.797 | 0.81 | 0.564 |
|  |  |  | (0.47, 1.78) |  | (0.39, 1.67) |  |
| Quartile 3 | 9 (6.0) | 12 (8.1) | 0.63 | 0.309 | 0.55 | 0.266 |
|  |  |  | (0.26, 1.54) |  | (0.19, 1.58) |  |
| Quartile 4 | 8 (5.4) | 22 (14.8) | 0.31 | 0.009 | 0.20 | 0.002 |
|  |  |  | (0.13, 0.75) |  | (0.07, 0.56) |  |

^1^Adjusting for all variables listed in Table 1 except age and gender.

^2^90- and 270-day drug exposure windows were defined as the use of study drug within 90 days and 270 days, respectively, from the date of first pharmacy claim after 90-day and 270-day, respectively, complete treatment period
